# Transcriptomic–proteomic analysis reveals the regulatory mechanisms of Alfalfa (*Medicago sativa*) in response to *Fusarium acuminatum*

**DOI:** 10.3389/fpls.2025.1620189

**Published:** 2025-07-29

**Authors:** Zhidan Shi, Hongxia Sun, Lifen Hao, Yongqing Yang, Chen Guo, Haiyan Huangfu, Hongli Huo, Lili Zhao, Jiuru Huangfu, Haijun Ding, Yongyu Fang, Ziqin Li, Yiding Niu

**Affiliations:** ^1^ College of Life Sciences, Inner Mongolia University, Hohhot, China; ^2^ Grassland Research Institute, Inner Mongolia Academy of Agricultural & Animal Husbandry Sciences, Hohhot, China; ^3^ Institute of Grassland Research, Chinese Academy of Agricultural Sciences, Hohhot, China

**Keywords:** *Fusarium* root rot, proteome, transcriptome, plant-pathogen interaction, association analysis

## Abstract

Alfalfa (*Medicago sativa*) is the most widely cultivated and important forage crop worldwide, owing to its high protein content. However, alfalfa *Fusarium* root rot seriously affects and restricts the yield and quality. This study explores the response mechanism of alfalfa to *Fusarium* root rot. we used transcriptomic and proteomic methods to analyze differentially expressed genes (DEGs) and differentially expressed proteins (DEPs) in alfalfa inoculated with *Fusarium acuminatum* on 0, 3, and 13 days. In total, 13,017 DEGs (6902 upregulated, 6115 downregulated) and 4830 DEPs (697 upregulated, 659 downregulated) were identified. Gene Ontology (GO) and Kyoto and Encyclopedia of Genes and Genomes (KEGG) analyses indicated that the DEGs and DEPs were mainly enriched in the cell cortex, flavonoid biosynthesis, and amino acid metabolism on day 3, whereas on day 13, they were primarily enriched in the cell wall, defense response, and flavonoid biosynthesis. Transcriptome and proteome analyses showed the same expression pattern of 81 genes and their corresponding proteins, which were mainly enriched in amino acid metabolism, cell wall synthesis, flavonoid biosynthesis, glucose metabolism, and plant-pathogen interactions. The results indicate that when alfalfa responds to infection by *F. acuminatum*, cell walls, antioxidant and defense-related enzymes, as well as genes involved in glucose metabolism and disease resistance, play important roles.This study contributes to the understanding of the molecular mechanism of the alfalfa response to *F. acuminatum* infection and provides an important basis for further research and in-depth characterization of candidate genes for breeding alfalfa root rot resistance.

## 1 Introduction

Alfalfa is a perennial leguminous forage grass that occupies an important position in animal husbandry because of its high yield and quality. It is widely used in feed production. In China, with the implementation of a series of policies to support the cultivation of alfalfa and other forage products, the alfalfa industry has achieved unprecedented development, with the planting area continuing to expand. However, many alfalfa diseases seriously restrict the healthy development of the alfalfa industry. The most representative is alfalfa root rot (*Fusarium* root rot) (Fang et al., 2019; Guo et al., 2022). According to statistics, the annual loss of alfalfa production caused by root rot worldwide is approximately 20%, and in severe cases, it is as high as 40%. Alfalfa root rot generally occurs in the main alfalfa planting areas in northwest China (Gansu, Xinjiang, Qinghai, etc.), northern China (Hebei and Inner Mongolia, etc.), and northeast China (Heilongjiang and Jilin, etc.), and the mortality rate of alfalfa in severe fields is > 60% (Li-xia et al., 2008; Pan et al., 2015). Alfalfa root rot is widely distributed and destructive and is one of the main factors leading to a decline in alfalfa yield and quality deterioration (Gao, 2024). This disease is usually caused by a combination of soil fungi, among which *Fusarium* fungi are the main pathogens causing root rot in alfalfa fields (Li et al., 2019). The incidence of *Fusarium* root rot in alfalfa has recently increased. To date, 25 species of *Fusarium* have been reported to cause root rot in alfalfa. There are eight main *Fusarium* species causing alfalfa root rot in China (Fang et al., 2019), namely, *Fusarium oxysporum*, *Fusarium solani*, *Fusarium acuminatum*, *Fusarium equiseti*, *Fusarium semitectum*, *Fusarium avenaceum*, *Fusarium proliferatum*, *and Fusarium tricinctum*. In recent years, it has been reported in China that *Fusarium incarnatum*, *Fusarium redolens*, *Fusarium verticillioides*, *Fusarium falciforme*, and *Fusarium virguliforme* can also cause alfalfa root rot (Wang et al., 2023; Yang et al., 2024). Therefore, it is of great significance to study the molecular mechanisms of alfalfa in response to *Fusarium* infection to formulate effective prevention and control strategies for alfalfa root rot.

During *Fusarium* infestations, plants produce complex physiological responses. For example, previous studies have found that proteins associated with photosynthesis are mostly inhibited, whereas pathways involved in energy metabolism, protein synthesis, and conversion are often upregulated (Cong et al., 2017; Wang et al., 2024). In these studies, the plants also exhibited other characteristic changes, including to their glucose metabolism, cell wall, reactive oxygen species (ROS), increased enzyme activity, expression of pathogenicity-related (PR) proteins, and secondary metabolites (Kang et al., 2019; Fang et al., 2023). Wang’s results indicate that after inoculation with Fusarium, the levels of soluble glucose, soluble protein, and malondialdehyde (MDA) in alfalfa plants changed significantly. The cytochrome P450, MYB, ERF, NAC, and bZIP protein families are also considered to be closely related to the resistance of alfalfa to root rot (Wang et al., 2023). In an experiment in which kidney bean roots were inoculated with *F. oxysporum*, transcriptome and metabolome analyses showed that the salicylic acid, jasmonic acid, and ethylene pathways were significantly enriched, with the enrichment of the flavonoid biosynthesis pathway being the most significant (Chen et al., 2019). In transgenic alfalfa plants with down-regulated expression of Caffeoyl*-CoA O-methyltransferase*(*CCoAOMT*) gene, the resistance to *Fusarium* wilt was enhanced. Transcriptomic and metabolomic analyses showed that the down-regulation of *CCoAOMT* led to the accumulation of (iso)flavonoids and intermediate compounds in their pathway, thereby improving the disease resistance of alfalfa (Gill et al., 2018). Protein expression plays an important role in plant responses to environmental stress and infections. A quantitative proteomic analysis of two rice genotypes 93-11 and Nipponbare, with different levels of disease resistance after inoculation with *Fusarium fujikuroi*, suggested that aquaporin PIP2-2 may play an antagonistic role against rice blast disease (Ji et al., 2019). The roots and leaves of alfalfa inoculated with *F. proliferatum* were analyzed by proteomics, revealing 66 DEPs in the roots and 27 DEPs in the leaves. These identified proteins were classified into different categories involving defense and stress response-related metabolism, photosynthesis, and protein synthesis (Cong et al., 2017).

Inner Mongolia, one of the main alfalfa planting areas in China, occupies an important position in the development of the alfalfa industry. The author collected alfalfa root rot samples from four major alfalfa-producing areas in Inner Mongolia, China, and identified the dominant pathogen as *F. acuminatum* (Sun, 2023). When Wang isolated and identified alfalfa root rot in different regions of Inner Mongolia, he found that the dominant pathogen was *F. acuminatum* (Wang, 2023). In recent years, several studies have shown that *F. acuminatum* can cause root rot in a variety of plants, including angelica, lentils, peppercorns, and astragalus (Zhang, 2021; Ruan et al., 2022; Bugingo et al., 2024; Mu et al., 2024). Current studies on *F. acuminatum* mostly focus on the isolation and identification of pathogens, determination of their pathogenicity and biological characteristics, evaluation of resistance to various pathogens, and biological control. The mechanism of the interaction between *F. acuminatum* and host plants has rarely been reported. Using alfalfa inoculated with *F. acuminatum* as experimental materials, we analyzed the changes in gene and protein expressions of alfalfa during the infection process of *F. acuminatum* using transcriptome and proteome sequencing. In the present study, we aimed to explore the molecular mechanisms of alfalfa resistance to *F. acuminatum* and identify candidate genes associated with resistance or susceptibility, which will not only provide a new perspective for the study of the molecular mechanisms underlying alfalfa resistance to *Fusarium* root rot but also establish a valuable genetic basis for the breeding of alfalfa disease-resistant varieties and contribute to the healthy and sustainable development of China’s alfalfa industry.

## 2 Materials and methods

### 2.1 Plant growth and fungal treatment

**Figure 1:**
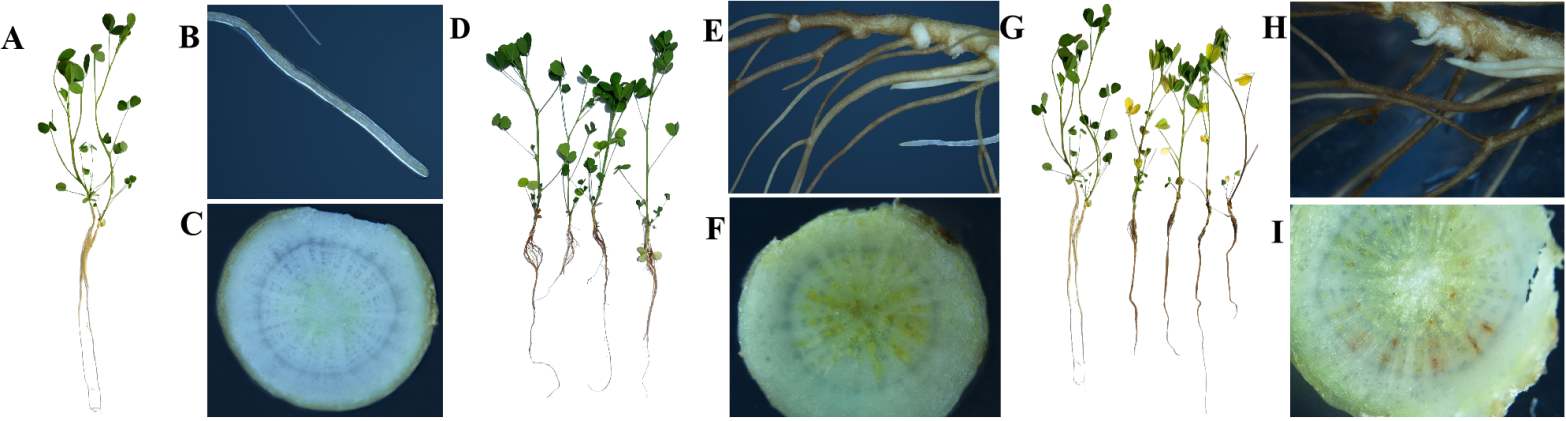
Phenotypic observation of alfalfa inoculated with *F. acuminatum*. **(A–C)** alfalfa root tissue infected with *F. acuminatum* for 0 days. **(D–F)** alfalfa root tissue infected with *F. acuminatum* for 3 days. **(G–I)** alfalfa root tissue infected with *F. acuminatum* for 13 days.

The *F. acuminatum* TZ-1 strain used in this study was isolated and preserved in the laboratory. The strain was cultured on potato glucose agar medium (PDA) for 5–7 days at 25°C and then transferred to carboxymethyl cellulose (CMC) media. The culture broth was incubated at 25°C, with shaking at 150 rpm, for 3 days. The spore concentration in the culture was calculated and used as the inoculum. Alfalfa seeds “Monopoly,” provided by the Grassland Research Institute at the Academy of Agricultural and Animal Husbandry Sciences of Inner Mongolia Autonomous Region, were selected as the experimental plants. The seeds were soaked in 5% bleach (sodium hypochlorite) solution for 3 min and 75% alcohol for 2 min, washed with distilled water four times, and then placed in a square box covered with gauze for germination. After 10 days, the seedlings were carefully removed from the gauze and transplanted onto a foam board with three seedlings per hole. The transplanted seedlings were moved into plastic hydroponic tanks (30 cm × 20 cm × 12 cm) filled with 7 L of nutrient solution. The photoperiod was set at a 16/8-h light/dark cycle at 25°C and 16°C, respectively. After 25 days of cultivation, the alfalfa seedlings were divided into two groups, with 27 plants in each group. The control group was cultured in a nutrient solution, and the treatment group was inoculated with *F. acuminatum* at a final concentration of 10^6^ spores/mL. Three biological replicates were used for each treatment group. The onset of symptoms was inspected periodically after inoculation (**Figure 1**). Alfalfa root samples (3–5 g) were collected at 0, 3, and 13 days post- inoculation (dpi) and placed in 15-mL centrifuge tubes. The samples were frozen in liquid nitrogen immediately after collection and divided into two parts. One portion was stored in a freezer at –80°C for subsequent transcriptome and proteomic analysis, and the other portion was used for enzyme activity detection.

### 2.2 Determination of physiological and biochemical indicators

To evaluate the physiological changes in alfalfa after inoculation with *F. acuminatum*, the activities of four key enzymes, including lipoxygenase (LOX), ascorbate peroxidase (APX), superoxide dismutase (SOD), and catalase (CAT), were determined at different time points. Enzyme activity assays were performed according to the manufacturer’s instructions (Suzhou Grace Bio-Technology Co., Ltd.) to ensure the accuracy and reproducibility of the experimental results.

### 2.3 RNA-seq transcriptome sequencing

Total RNA was extracted from the nine samples using the RNAprep Pure Plant Plus Kit (QIAGEN, Germany). The quality and concentration of RNA were determined by an Agilent 2100 bioanalyzer (Novogene, Wuhan, China) and a Qubit 2.0 fluorometer (Novogene, Wuhan, China) to ensure they met the requirements for library construction. The sequencing library was constructed by referring to the instruction manual of the NEBNext^®^ Ultra™ RNA Library Prep Kit for Illumina^®^. Transcriptome sequencing was performed after the library inspection. Sequencing was performed on an Illumina NovaSeq 6000 platform (Novogene, Wuhan, China). Raw sequencing data were filtered using fastp software (version 0.19.7) to obtain clean reads for subsequent analysis. Genomic data of Medicago sativa (https://figshare.com/projects/whole_genome_sequencing_and_assembly_of_Medicago_sativa/66380) and gene model annotation files were downloaded from the genome website. HISAT2 (v2.0.5) was used to construct indexes for the reference genome, and paired-end clean reads were aligned to the reference genome. StringTie (1.3.3b) was adopted for novel gene prediction. Subsequently, featureCounts (1.5.0-p3) was used to perform quantitative analysis of gene expression levels for each sample. DESeq2 software (1.20.0) was used for differential expression analysis of each comparison group. When the corrected p-value (padj) of a gene was < 0.05 and |log2(fold change)| ≥ 1, the gene was considered a DEG. Subsequently, the clusterProfiler software was used to conduct a GO functional enrichment analysis and KEGG pathway enrichment analysis on the DEG set, with padj ≤ 0.05 as the threshold for significant enrichment.

### 2.4 Proteomic analysis

The samples for proteome sequencing were the same as those for the transcriptome analysis, and Tandem Mass Tags (TMT) protein quantification was performed using Novogene (Wuhan, China). The main contents included the processes of protein extraction, quantification, detection, enzymatic digestion and desalination, labeling, the enrichment of modified peptides, separation of fractions, and mass spectrometry detection, all of which were carried out in accordance with Novogene’s standard procedures. The obtained mass spectrometry proteomics data have been uploaded to the iProX database with the PXD number PXD053965 (https://www.iprox.cn/page/project.html?id=IPX0009237000). Based on the raw files obtained following the mass spectrometry detection, database retrieval was conducted using Proteome Discoverer2.4 software to retain spectral peptides and reliable proteins (proteins containing at least one unique peptide segment [specific peptide segment]) with a credibility of more than 99%. False discovery rate (FDR) verification was performed to remove peptide segments and proteins with an FDR greater than 1%. After the database search had been completed, a series of quality controls were performed on the peptides and proteins. The identified proteins were functionally annotated using the GO, KEGG, and COG databases. The relative quantitative values of each protein in the two comparative samples were tested using a *t*-test, and the corresponding *P*-values were calculated. *P*-value ≤ 0.05 and the multiple of difference |log2(FoldChange)| ≥ 1.2 were the thresholds for screening for differential proteins.

### 2.5 Statistical analysis

The data obtained from physiological and biochemical indices experimental determinations were sorted and analyzed using the word processing system (Kingsoft Software Co., Ltd., Beijing, China). The mean square error test was performed using the Levene test in SPSS 22.0 statistical software (International Business Machines Co., Ltd., New York, NY, USA). When evaluating the differences between two groups, p < 0.05 was considered statistically significant. GraphPad 9 (GraphPad Software Inc., San Diego, CA, USA) was used for graphs plotting.

## 3 Results

### 3.1 Physiological analyses

**Figure 2:**
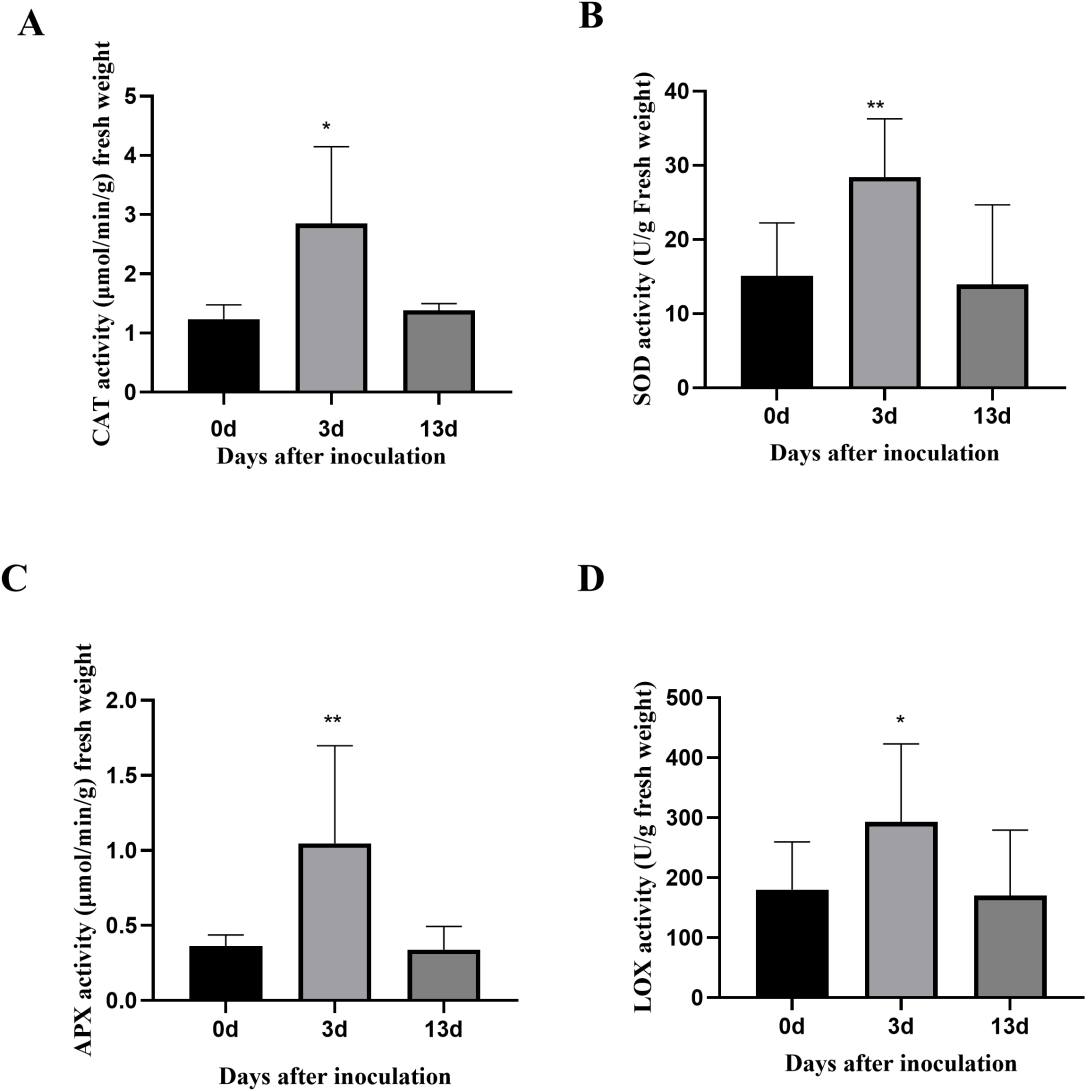
The activities of 4 key enzymes. The horizontal coordinate is the time of inoculation with *F. acuminatum*. The vertical coordinate is the activity data of enzyme. **(A)** The content of CAT: Catalase. **(B)** The content of SOD: Superoxide dismutase. **(C)** The content of APX: ascorbate peroxidase. **(D)** The content of LOX: Lipoxygenase. Each error bar represents the mean ± standard deviation of 3 repetitions. ** means p-value<0.01

To analyze the internal changes after inoculation with *F. acuminatum* in alfalfa, the contents of the four key enzymes were determined at different time points before and after inoculation. The results showed that 3 dpi, CAT activity in the alfalfa increased by 95%, SOD activity by 58%, APX activity by 6.5%, and LOX activity by 48%. When the inoculation period was extended to 13 days, the activities of CAT, SOD, APX, and LOX decreased, approaching the enzyme activity levels observed before inoculation (0 days) (**Figure 2**). These results indicate that during the defense response of alfalfa against *F. acuminatum*, the CAT response was the strongest at 3 dpi, followed by that of SOD and LOX, while APX exhibited the most stable response.

### 3.2 Transcriptome sequencing and correlation analysis

To elucidate the molecular transcriptional mechanisms of alfalfa in response to *F. acuminatum* infection, the transcriptomes of nine samples from the three treatment groups (0, 3, and 13 days) were sequenced. A total of 3.99 × 10^8^ raw reads were obtained (**Table 1**). The obtained Transcriptome data have been uploaded to the SRA database with accession number PRJNA1256159 (https://dataview.ncbi.nlm.nih.gov/object/PRJNA1256159?reviewer=4605p1u48mql5f47ik6iu58t9n). After quality control and the removal of low-quality reads, adapter contamination, and reads with an excessively high unknown base (N) content, 3.92 × 10^8^ high-quality clean reads were retained. The proportion of QC_20_ reads exceeded 96%, whereas that of QC_30_ reads exceeded 90%, with the GC content ranged from 39–41%. The mapping rate of clean reads to the designated reference genome was > 82% for each sample. The Pearson correlation coefficient among biological replicates was greater than 0.8 (**Supplementary Figure S1**), indicating high sequencing quality, good intragroup reproducibility, and reliable data suitable for a DEG analysis.

**Table 1:** Sequencing data statistics.

Sample	Raw reads	Clean reads	Clean bases	Error rate	Q30	GC content
0d-1	45377844	43738466	6.56G	0.03	91.79%	41.78%
0d-2	43439532	42428960	6.36G	0.03	91.89%	41.72%
0d-3	45592020	44592508	6.69G	0.03	91.17%	39.79%
3d-1	45736368	45054298	6.76G	0.03	91.64%	41.27%
3d-2	45686130	44685580	6.7G	0.03	92.04%	41.44%
3d-3	42594746	42016914	6.3G	0.03	92.09%	41.42%
13d-1	40545528	39936974	5.99G	0.03	90.76%	41.29%
13d-2	45868334	45126668	6.77G	0.03	90.71%	41.32%
13d-3	45022256	44495108	6.67G	0.03	91%	41.85%

Sample, sample analysis number of alfalfa root; Raw reads, Number of original reads; clean reads, the numbers of pair-end reads in clean data; clean bases, total number of bases; Error rate, Data overall sequencing error rate; Q30, the percentage of bases with a mass greater than or equal to 30; GC content, the percentage of G and C bases in clean data.

### 3.3 Analysis of the DEGs

**Figure 3:**
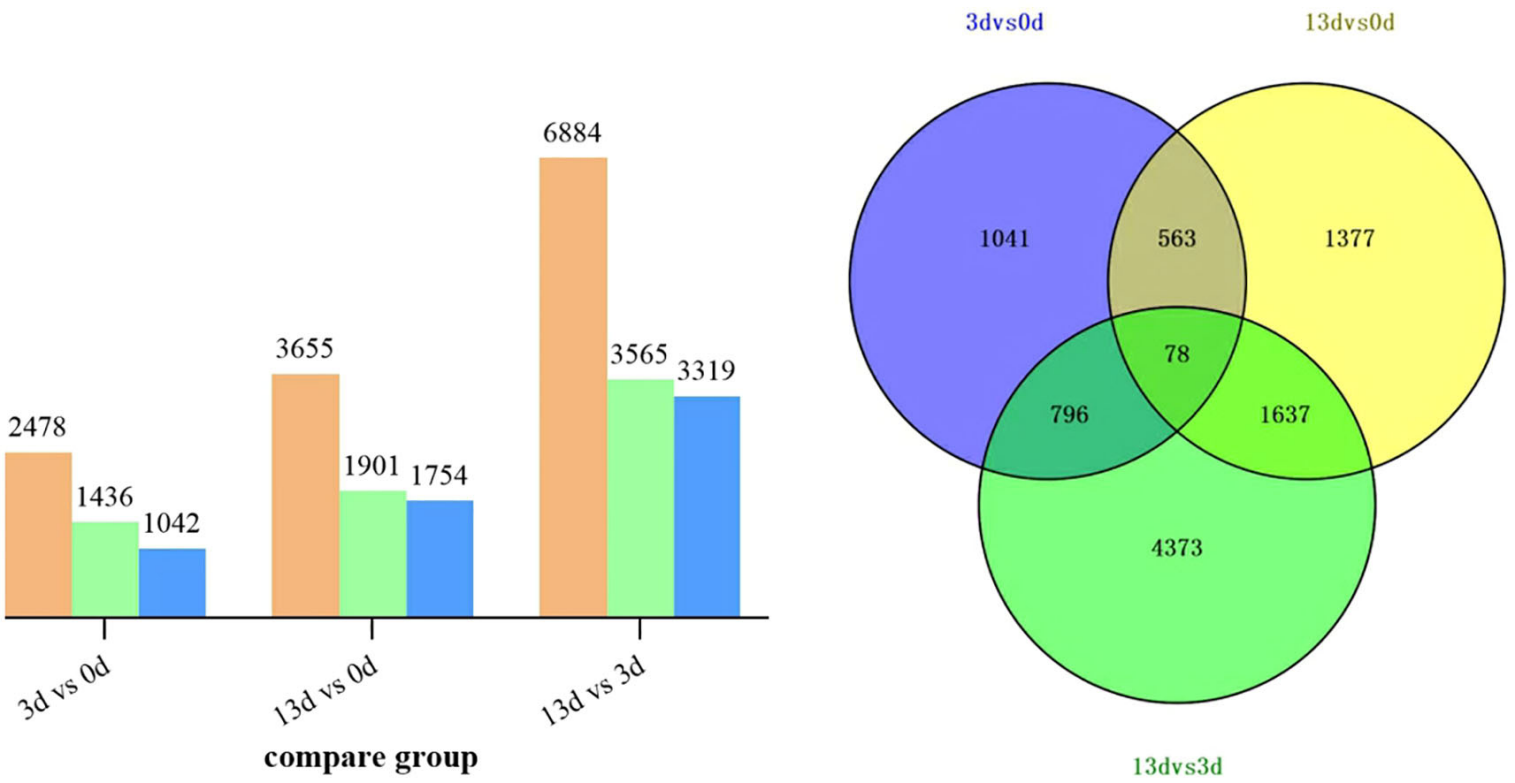
DEGs in alfalfa roots infected by *F acuminatum*. **(A)** Orange represents the total number of DEGs, green represents upregulation and blue represents downregulation. **(B)** Overlap of differential genes between different comparison combinations.

To clarify the changes in related genes in alfalfa roots infected with F. acuminatum, we performed a DEG analysis. A total of 13017 DEGs were obtained. Compared with 0 days, 2478 genes showed expression changes at 3 days after inoculation, among which 1436 genes were up-regulated and 1042 genes were down-regulated. When13 days after inoculation was compared with 0 days, 3655 genes showed differential expression, including 1901 up-regulated genes and 1754 down-regulated genes. When comparing 13 days with 3 days after inoculation, a total of 6884 DEGs were detected, of which 3565 were up-regulated and 3319 were down-regulated (**Figure 3A**). A Venn diagram analysis (**Figure 3B**) revealed that within 13 days of *F. acuminatum* inoculation in alfalfa roots, 78 genes were differentially expressed throughout the infection period. A total of 9865 DEGs were identified during the infection process, with 4373 (44.33%) DEGs specifically expressed between days 3 and 13, indicating that the alfalfa transcriptome changed significantly after 3 days of inoculation.

**Figure 4:**
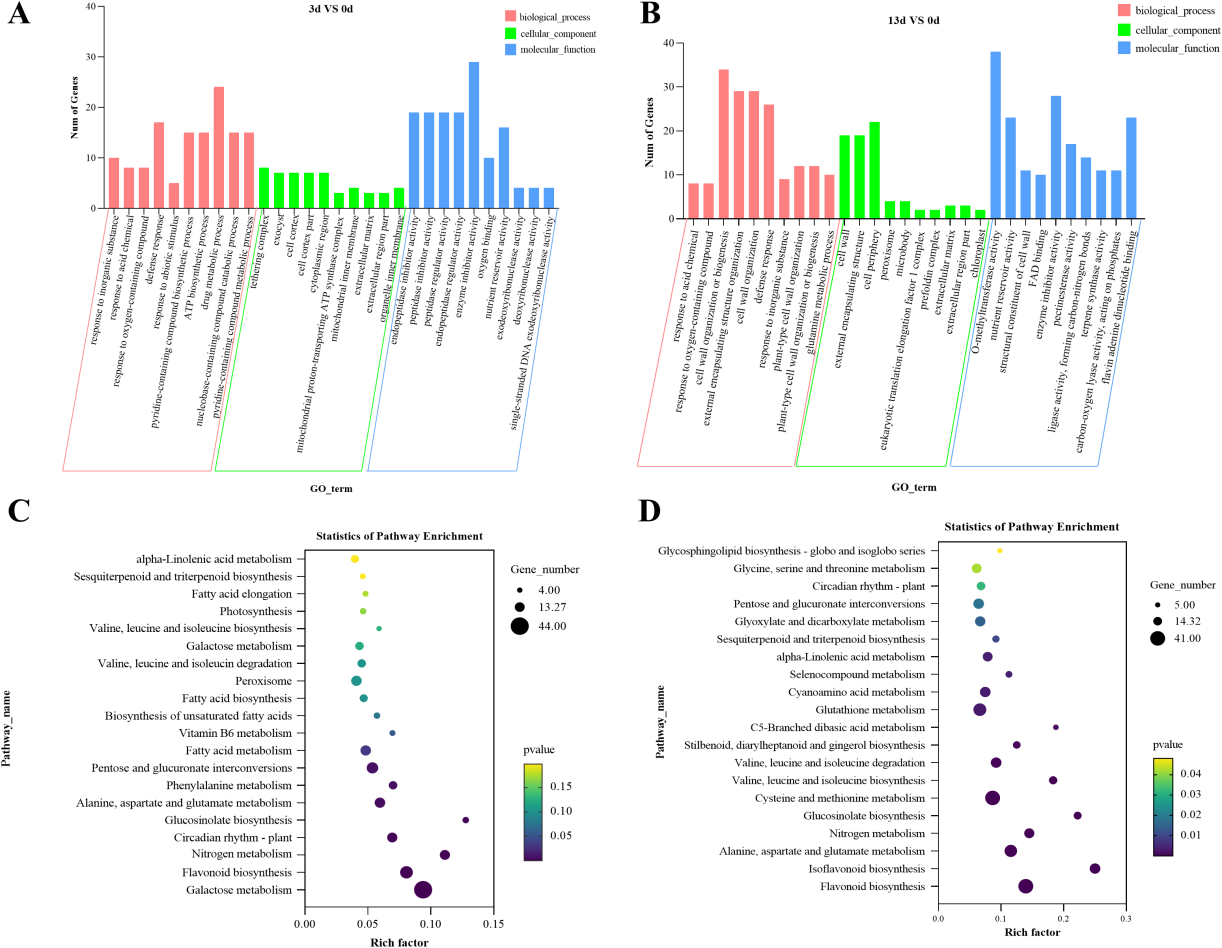
GO classification and KEGG enrichment analysis of DEGs in alfalfa inoculated with *F acuminatum*, sampled at 0 days, 3 days, and 13 days post inoculation, **(A, B)** GO classification of DEGs, and **(C, D)** KEGG enrichment analysis of DEGs.

To understand the potential pathways and functions of these DEGs in the alfalfa response to *F. acuminatum*, we performed GO and KEGG enrichment analyses. The GO analysis of the above DEGs indicated that 3 days after inoculation with the pathogen, the DEGs were mainly enriched in functional units such as drug metabolic process, defense response, tethering complex, cell cortex, cell cortex part, enzyme inhibitor activity, and peptidase regulator activity (**Figure 4A**). Thirteen days after inoculation with the pathogen, the DEGs were mainly enriched in functional units, such as cell wall organization or biogenesis, cell wall organization, defense response, cell periphery, cell wall, O-methyltransferase activity, enzyme inhibitor activity, and nutrient reservoir activity (**Figure 4B**). A comparative analysis of the GO enrichment results of DEGs at 3 and 13 days after *F. acuminatum* inoculation revealed that during the resistance of alfalfa to the pathogen, functional units such as cell wall organization, defense response, and enzyme inhibitor activity played significant roles.

To determine the signaling pathways involved, a KEGG enrichment analysis was performed on the DEGs. Three days after inoculation, the DEGs were mainly enriched in the following pathways: glutathione metabolism (mtr00480); flavonoid biosynthesis (mtr00941); pentose and glucuronate interconversions (mtr00040); alanine, aspartate, and glutamate metabolism (mtr00250); and fatty acid metabolism (mtr01212) (**Figure 4C**). Thirteen days after inoculation, the DEGs were mainly enriched in the following pathways: flavonoid biosynthesis (mtr00941); cysteine and methionine metabolism (mtr00270); glutathione metabolism (mtr00480); alanine, aspartate, and glutamate metabolism; and isoflavonoid biosynthesis (mtr00943) (**Figure 4D**). At both 3 and 13 dpi, the pathways of flavonoid biosynthesis, glutathione metabolism, and amino acid metabolism were significantly enriched, indicating that these pathways are closely related to the resistance of alfalfa to infection.

### 3.4 Proteome sequencing

**Figure 5:**
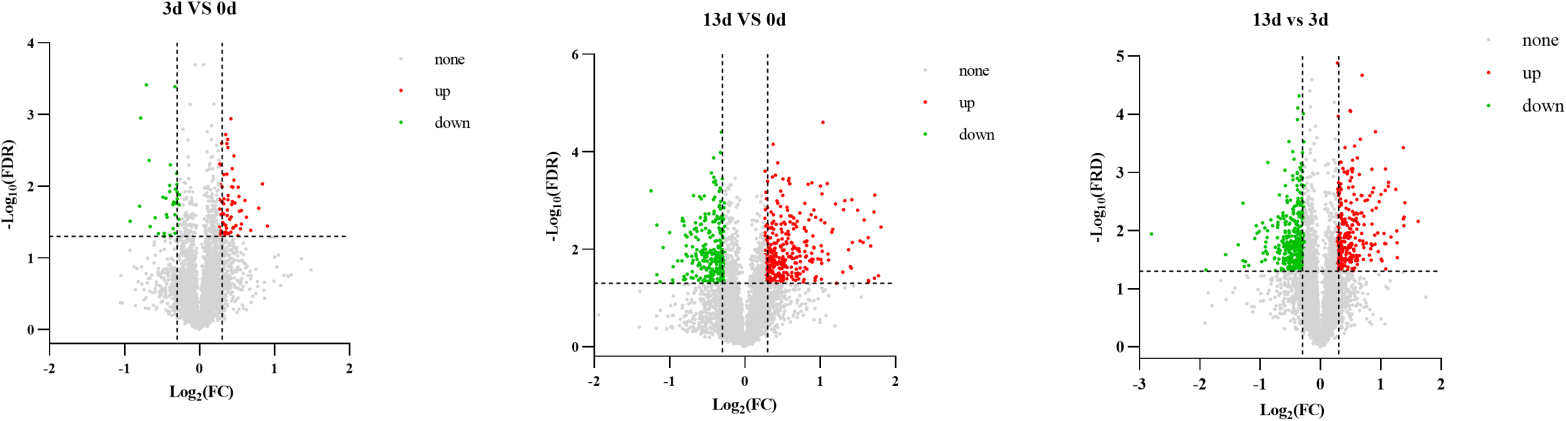
Volcano plot of DEPs. Each point in the differential expression volcano map represents a protein, and the abscissa represents the logarithm of the differential expression of the protein in the samples. The ordinate represents the negative logarithm of the statistically significant changes in protein expression. The green and red dots represent downregulated and upregulated protein, respectively.

To further explore the regulatory effects of root rot on DEPs in alfalfa, TMT technology was used to sequence and analyze pathogen-inoculated samples. A cumulative diagram analysis of the Coefficient of Variance (CV) values for all proteins in the samples (**Supplementary Figure S2**) showed good experimental repeatability, ensuring reliable results. We obtained 23852 peptides from the proteome sequencing results of the nine samples and identified 4847 proteins, of which 4830 were quantified. Based on the proteome data, further differential protein analyses were carried out at different inoculation time points. The results showed that compared with 0 days, after inoculation with the pathogen in the alfalfa roots for 3 days, a total of 103 DEPs were detected, among which 67 were up-regulated and 36 were down-regulated. After 13 days of inoculation, 640 DEPs were detected, of which 361 were upregulated and 279 were downregulated. Compared with 3 days after inoculation, a total of 613 DEPs were detected at 13 days, of which 269 were up-regulated and 344 were down-regulated (**Figure 5**).

### 3.5 Analysis of the DEPs

**Figure 6:**
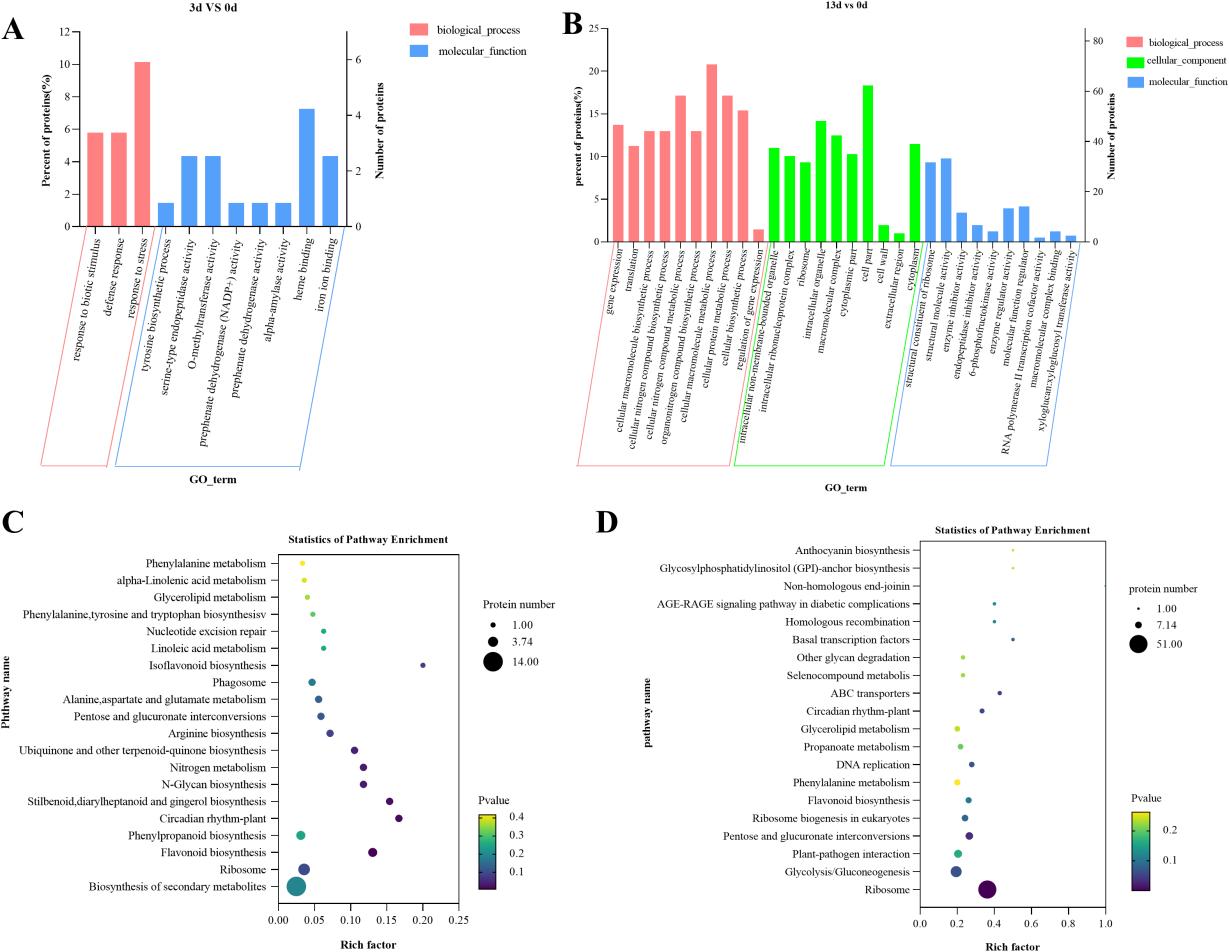
GO classification and KEGG enrichment analysis of DEPs in alfalfa inoculated with *F acuminatum*, sampled at 0 days, 3 days, and 13 days post inoculation, **(A, B)** GO classification of DEPs, and **(C, D)** KEGG enrichment analysis of DEPs.

To further analyze the resistance mechanism of alfalfa to *Fusarium* root rot, we performed GO and KEGG enrichment analyses on the selected DEPs. In the GO enrichment analysis, after 3 days of inoculation, the DEPs were significantly enriched in processes such as response to biotic stimulus (GO:0009607), defense response (GO:0006952), response to stress (GO:0006950), heme binding (GO:0020037), O-methyltransferase activity (GO:0008171), and iron ion binding (GO:0005506) (**Figure 6A**). This suggests that, during the early stages of infection, alfalfa mainly activates functions related to defense and stress responses.

After 13 days of inoculation, the enrichment of the DEPs showed a clear shift, with a significant focus on processes related to biological processes, such as cellular macromolecule metabolic processes (GO:0044260), cellular nitrogen compound metabolic processes (GO:0034641), and cellular protein metabolic processes (GO:0044267). Processes related to cellular components, such as cell parts (GO:0044464), intracellular organelles (GO:0043229), and macromolecular complexes (GO:0032991), as well as molecular functions such as structural molecule activity (GO:0005198), structural constituents of ribosomes (GO:0003735), and enzyme regulator activity (GO:0030234), were also enriched (**Figure 6B**). These results indicate that, as the infection progresses, alfalfa activates functions related to cellular metabolism and structural maintenance.

The KEGG enrichment analysis results showed that after 3 days of inoculation, the DEPs were primarily enriched in the biosynthesis of secondary metabolites (map01110), ribosomes (map03010), flavonoids (map00941), and phenylpropanoids (map00940) (**Figure 6C**). After 13 days of inoculation, the DEPs were mainly enriched in the ribosome (map03010), glycolysis/gluconeogenesis (map00010), and plant-pathogen interaction (map04626) pathways (**Figure 6D**). This indicates that alfalfa initially defends against the pathogen *F. acuminatum* by synthesizing secondary metabolites and later responds through energy metabolism and the direct defense against fungal infection.

### 3.6 Correlation analysis of the transcriptome and proteome sequencing

The relationship between mRNA and protein expression is complex. To elucidate the co-expression in alfalfa at the protein and transcriptional levels during *F. acuminatum* infection, we performed a joint analysis of alfalfa transcriptome and proteomic data. A total of 124 DEGs and 439 DEPs were detected in the 3 days vs. 0 days groups (**Supplementary Table S1**), of which 24 were significantly different at both the transcript and protein levels (18 were downregulated and 2 were upregulated). A total of 201 DEGs and 563 DEPs were examined in the 13 days vs. 0 days group (**Supplementary Table S2**), of which 35 were significantly different at the transcript and protein levels (13 downregulated and 18 upregulated). A total of 453 DEGs and 605 DEPs were examined in the 13 days vs. 3 days group (**Supplementary Table S3**), of which 71 were significantly different at the transcript and protein levels (6 were downregulated and 24 were upregulated). A GO functional classification of the significant proteins and transcripts with consistent expression trends was performed. The results showed that fewer functions were significantly enriched after 3 days, whereas more functions were significantly enriched after 13 days, including macromolecular metabolic processes, amino acid metabolism, cell wall organization, various enzyme activities, and stimulus responses. To further explore the biological pathways through which DEGs and proteins exert their effects, we performed a KEGG enrichment analysis on these significantly expressed proteins and transcripts with consistent expression trends. Metabolic pathways such as secondary metabolite biosynthesis (MAP01110), phenylpropanoid biosynthesis (MAP00940), and nitrogen metabolism (MAP00910) were significantly enriched after 3 days. On day 13, pentose and gluconic acid interconversion (MAP00040), flavonoid biosynthesis (MAP00941), ABC transporter (MAP02010), galactose metabolism (MAP00052), glycolysis/gluconeogenesis (MAP00010), and plant-pathogen interaction (MAP04626) were significantly enriched. These results indicate that in the early response of alfalfa to infection, the expression of genes related to secondary metabolism and nitrogen metabolism pathways is involved, and more pathways are involved, including the expression of key genes related to flavonoid biosynthesis, glucose metabolism, plant disease resistance, and secondary metabolic pathways.

#### 3.6.1 Proteins in the plant-pathogen interaction

**Figure 7:**
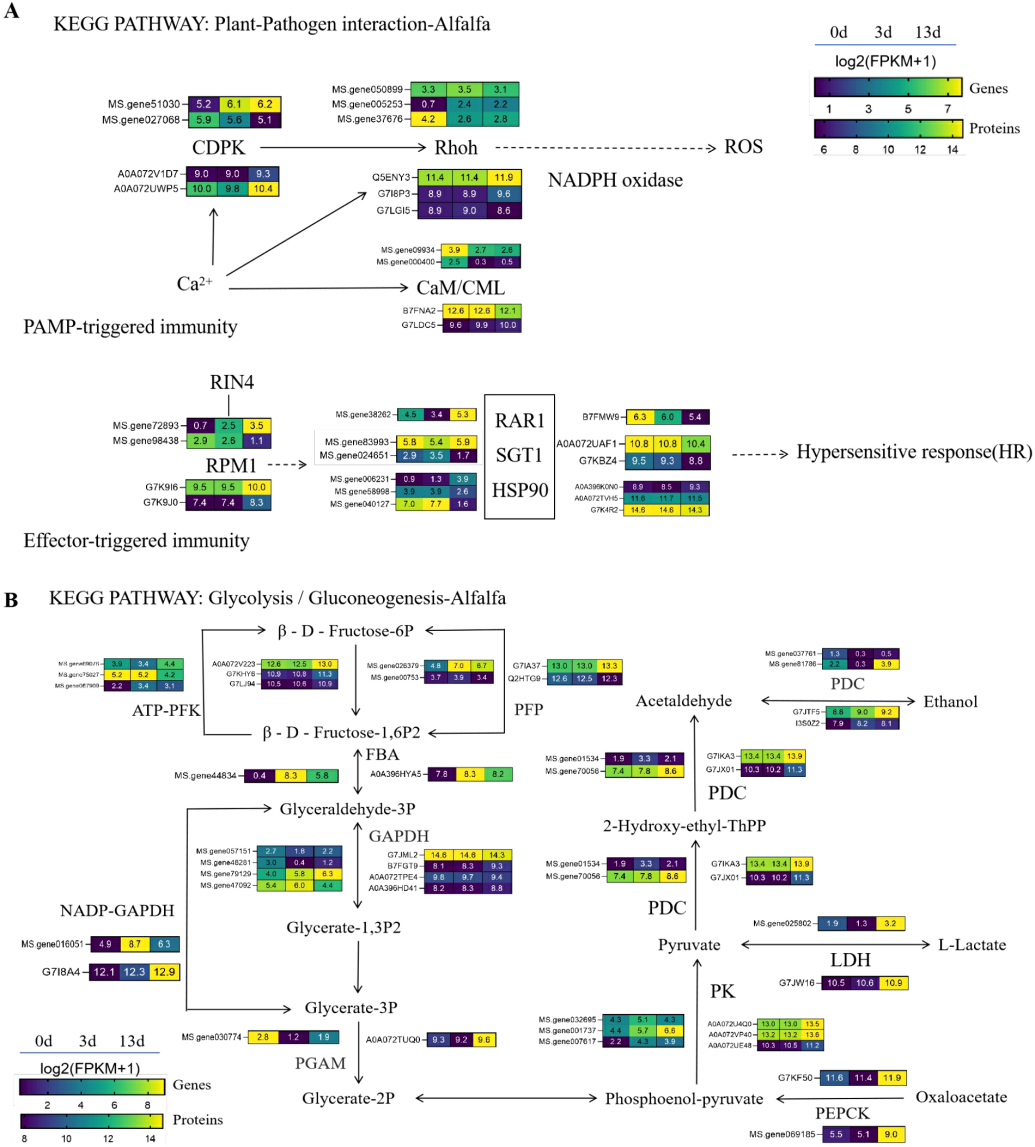
The important pathway for alfalfa to respond to *F acuminatum*. This pathway was constructed based on the KEGG pathway. Levels of genes and proteins were averaged over three biological replicates. The color scale represents the value of log2(FPKM+1) in three groups. **(A)** The relative changes of key genes and proteins in the plant-pathogen interaction pathway. **(B)** The relative changes of key genes and proteins in Glycolysis/Gluconeogenesis pathway.

In the proteomic analysis, we identified 15 DEPs associated with plant-pathogen interaction pathways (**Figure 7A**), which were also identified in the transcriptome. Among these DEPs, two belonged to disease resistance proteins and three belonged to the RBOH family, both of which were upregulated in the 13 days vs. 0 days groups. Three heat shock protein (Hsp)-90 family proteins and their co-chaperones, two SGT family proteins, were also identified: one Hsp90 protein was upregulated, whereas the other was downregulated in the 13 days vs. 0 days groups, and all SGT proteins were downregulated. The downregulation of RAR1 was also observed. Among the four 4 EF hand calcium-binding family proteins, two belonged to the calcium/calmodulin-dependent protein kinase (CDPK) family. The expression of these CDPK family members varied, and upregulation was observed in the 13 days vs. 0 days groups. These results suggest that the CC-NBS-LRR disease resistance genes, ROS, calcium signaling pathways, and other cellular signaling pathways are important routes for alfalfa to respond to *F*. *acuminatum*.

#### 3.6.2 Proteins in the glycolysis/gluconeogenesis pathway

Twenty-one DEPs were identified in the glycolysis/glucose metabolism pathway (**Figure 7B**). Only two DEPs were identified in the 3 days group, both of which were upregulated. In the 13 days group, 19 DEPs were identified, including 5 belonging to the phosphofructokinase type A family (PFK-A), with most showing upregulation. Four DEPs belonged to the GAPD (glyceraldehyde-3-phosphate dehydrogenase) family, including two upregulated and two downregulated genes. These three DEPs belonged to the pyruvate kinase family, all of which were upregulated. Additionally, the identified PGAM (phosphoglycerate mutase), PEPC (phosphoenolpyruvate carboxylase, EC 4.1.1.31), TPP (trehalose-6-phosphate phosphatase), LDH (lactate dehydrogenase, EC 1.1.1.27), and ADH (alcohol dehydrogenase, EC 1.1.1.1) are all key enzymes in the glucose metabolism process, and all were upregulated. These results indicate that glucose signaling participates in the signal transduction pathways of alfalfa in response to *F. acuminatum*.

## 4 Discussion

Plants have evolved complex stress-resistance mechanisms to cope with harsh natural conditions as they grow (Glazebrook, 2005; Höfte and Voxeur, 2017). The plant cell wall is the first physical barrier against pathogen invasion and is often thought to be a passive barrier initiated during pathogen invasion. However, an increasing number of studies have suggested that the cell wall may actively play a defensive role (Scheller and Ulvskov, 2010; Wan et al., 2021). Cell walls undergo structural changes as plants grow, develop, and respond to stress. As an important monitoring system, they undergo dynamic remodeling during adaptation to external stress from pathogens. Thus, maintaining cell wall integrity (CWI) is important in activating and monitoring plant defense responses (Zhao and Dixon, 2014; Vaahtera et al., 2019). In pathogen-associated molecular pattern-triggered immunity (PTI), the host exhibits cell wall thickening, lignification, phytoprotective hormone production, and PR gene expression (Liu et al., 2018). In addition, cellulose, dextrin, galacto-oligosaccharide, xylo-oligosaccharides, and substances released when the cell wall is infected with pathogens can act as effective elicitors or signaling molecules to enhance plant disease resistance (Wan et al., 2021). Zhao conducted a comprehensive analysis using metabolomics, proteomics, and transcriptomics to identify the genes affecting the total lignin content in *Populus tomentosa*. Through GO and KEGG analyses of lignin-related modules, it was found that the total lignin content is influenced not only by individual lignin genes but also by genes involved in pathways such as “glutathione metabolic process,” “cellular modified amino acid metabolic process,” and “carbohydrate catabolic process” (Zhao et al., 2023). Glutathione peroxidase (GSH-PX) is involved in ROS and lignin metabolism and can significantly affect the content of total phenols as a precursor of lignin synthesis, as well as phenylalanine aminolyase (monolignol biosynthesis), thus affecting the total lignin content (Juan-juan et al., 2011). In the present study, the GO analysis of alfalfa DEGs showed that most of the alfalfa DEGs were enriched in the cell cortex, cell wall organization, and cell wall during infection with *F. acuminatum*. The results of the KEGG enrichment analysis showed that the DEGs were mainly enriched in glutathione metabolism (mtr00480), including 25 genes that were upregulated and 6 genes that were downregulated. Glutathione metabolism was related to the lignin content, indicating that these genes may play a role in cell wall morphological changes by affecting lignin synthesis. Pectin is an important component of the cell wall (Wan et al., 2021), and methylesterases can directly or indirectly participate in defense reactions by regulating the degree of the methyl esterification of pectin. Previous proteomics studies on *F. oxysporum*-infected resistant and susceptible chickpea genotypes have shown that methylesterase levels increased in both chickpea varieties 72 h after inoculation. However, methylesterase accumulated to a greater extent in the resistant varieties (Chatterjee et al., 2014). Similarly, in the proteomic analysis of this study, it was found that the methyl esterase (G7K9E3) in alfalfa was significantly upregulated 3 days after pathogen inoculation. The GO analysis of DEGs in the transcriptome also showed that numerous genes were enriched in O-methyltransferase activity. The accumulation of methyl esterase may potentially be involved in cell wall repair. These results show that lignin and pectin, which are important components of the cell wall, play important defensive roles when alfalfa is infection by *F. acuminatum*.

Glucose in plants not only serves as an essential component for energy supply and structural building blocks but also possesses signal transduction functions, enabling it to regulate the expression of related genes and the activity of enzymes (Proels and Hückelhoven, 2014; He et al., 2016). Glucose can directly act as an antioxidant to quench oxidative substances (Kunz et al., 2014) and participate in plant growth and development, as well as adverse environmental responses (Lastdrager et al., 2014; Lu et al., 2015). Numerous studies have revealed the crucial role of glucose in the plant response to pathogenic stress. Wang et al. (2023) found that after inoculation with *F. acuminatum*, the soluble glucose content in both disease-resistant and disease-susceptible alfalfa varieties increased significantly, with the content in the resistant variety being approximately twice that in the susceptible one (Wang et al., 2023). Similarly, in the response of chickpeas to *F. oxysporum*, proteins involved in glycolysis showed an upward trend in their expression in disease-resistant varieties and a downward trend in susceptible varieties, indicating that the activation and rapid accumulation of sugars affect the sensitivity of chickpeas to pathogenic bacteria (Kumar et al., 2016). In addition, an omics analysis of *F. oxysporum* infection in common beans revealed that the amino sugar and nucleotide sugar metabolic pathways were significantly enriched (Chen et al., 2019). Collectively, these studies indicate that glucose metabolism is closely associated with plant disease resistance. The present study further elucidated the role of glucose in alfalfa resistance against *F. acuminatum* using omics analyses. The results showed that the proteins involved in the glycolytic pathway were significantly enriched. Trehalose, a typical stress metabolite, was significantly upregulated in the glycolytic pathway. Trehalose usually does not accumulate under suitable conditions but rapidly accumulates under stress (Tang et al., 2018; Wang et al., 2022). In plants, trehalose is synthesized via the TPS-TPP pathway (Vandesteene et al., 2012). In-depth analyses revealed that trehalose-6-phosphate phosphatase (TPP) was significantly upregulated among the DEPs involved in the glycolysis/glucose metabolism pathway. The GO analysis showed that the trehalose-6-phosphate (T6P) protein (A0A072U2F7) in the trehalose biosynthetic process was also significantly upregulated. Notably, previous studies have indicated that the trehalose metabolic pathway, which is regulated solely by the expression of trehalose-6-phosphate synthase (TPS), can only lead to a small amount of trehalose accumulation, which is insufficient to enhance plant resistance to osmotic stress. It has been speculated that a slight increase in trehalose may be a response to adverse stress as a signaling substance (Bischof, 2020). Other studies have shown that in the trehalose metabolic pathway, the change in the T6P content is more crucial for enhancing plant stress resistance (Paul et al., 2018). Previous studies on trehalose have mostly focused on aspects such as the abiotic stress response of plants and regulation of flowering time (Du et al., 2023). Combined with the fact that no significant upregulation of the TPS gene was detected in the transcriptome in the present study, but both the TPP and T6P proteins were significantly upregulated in the proteome, it is speculated that in the process of alfalfa coping with *F. acuminatum* stress, it is not trehalose itself but T6P that plays a dominant role in the trehalose metabolic pathway.

When plants are stressed by pathogenic bacteria, the antioxidant enzyme system indirectly enhances disease resistance by eliminating ROS. In this study, the antioxidant and defense-related enzymes (SOD, CAT, APX, and LOX) in alfalfa at different stages of inoculation were examined, including their activities, expression levels of their encoding genes, and protein abundance. The results showed that the activities of the SOD, CAT, APX, and LOX enzymes increased significantly after 3 days inoculation and decreased after 13 days inoculation. The expression of the gene encoding SOD (MS. gene013412) increased significantly after 3 days inoculation and decreased after 13 days inoculation. In contrast, the expression of CAT (MS. gene064838) and APX (MS. gene057684 and MS. gene071408) continued to increase after 3 days inoculation. Although the expression of the related genes changed, there was no significant difference in their protein expression. It has been speculated that this phenomenon may be caused by a post-transcriptional regulatory mechanism. As the main ROS scavenger in plants under stress, As a major ROS scavenger in plants under stress, SOD was abundantly expressed at 3 days after alfalfa inoculation with *F. acuminatum*. It can catalyze the disproportionation of reactive oxygen species and free radicals to generate H_2_O_2_ (Vandesteene et al., 2012). With the continuous accumulation of H_2_O_2_ during infection, APX and CAT are required to further convert it into H_2_O_2_ and O_2_, thereby reducing the toxicity of H_2_O_2_ to alfalfa cells and maintaining intracellular redox balance (Bischof, 2020). LOX, a class of versatile dioxygenases, plays a unique role in plant defense systems. It can catalyze the oxygenation of polyunsaturated fatty acids to produce hydroperoxides. Through the hydroperoxidation of linoleic and linolenic acids, precursors for jasmonic acid synthesis are produced (Moraga-Suazo et al., 2024), which, in turn, trigger the expression of plant defense-related genes (Yang et al., 2019). This study found that 12 LOX genes were significantly expressed in alfalfa, of which 9 were upregulated and 3 were downregulated. Among the corresponding proteins, three (G7KYM9 was upregulated, G7LIY0 was downregulated, and G7LIZ7 was downregulated) showed significant changes in expression, while the others showed no significant differences. Notably, the three significantly expressed LOX enzymes are involved in linoleic acid metabolism (map00591). Previous studies have shown that LOX expression in wheat is induced by rust disease and may be involved in the effector-triggered immunity (ETI) response (Gan et al., 2024). Maize *ZmLOX3* inhibits the colonization and sporulation of *Aspergillus flavus*. While the maize *LOX3* gene defends against the invasion of *Aspergillus flavus*, the *LOX* genes in *Aspergillus flavus* also participate in the host infection process and interact with the host *LOX* to participate in the host’s resistance and susceptibility to pathogenic fungi. *LOX* genes are important signal communication molecules in the interaction between the host and pathogenic fungi (Liu, 2016). In a previous study, the leaves of *Arabidopsis thaliana* and *Medicago sativa* overexpressing *MtLOX24* were infected with *Pseudomonas syringae pathovar tomato* DC3000 and *Fusarium chlamydosporum*. The degree of damage to the overexpression group was significantly less than that to the wild-type group (Xu, 2024). Therefore, the role of LOX in alfalfa resistance to *F. acuminatum* requires further in-depth study.

We further observed the significant enrichment of various receptor protein kinases and heat shock proteins (HSPs). Transcriptome analyses identified 61 HSP genes (27 upregulated and 34 downregulated) and 55 calcium-dependent protein kinase (CDPK) genes (7 upregulated and 48 downregulated). Thirteen HSPs (two HSP90s, two HSP70s, six HSP20s, and two other HSPs) and 20 CDPKs were identified through a proteomic analysis, of which three CDPKs were significantly expressed. HSPs are an important class of highly conserved and abundant molecular chaperones in most organisms. They exert a natural protective effect against various stressors by regulating the establishment of the tertiary structure of proteins, assembly of protein complexes, and transmembrane transport of proteins after processing (Jiang et al., 2023). During plant disease resistance, the interactions among HSP90, SGT1, and RAR1 play a crucial role in stabilizing the activity of R proteins and enabling plants to recognize pathogens. SGT1 interacts with the ATPase region and C terminus of HSP90 via its CS and TPR regions, respectively. The complex formed by these three components plays a central role in the plant disease resistance response triggered by R proteins (Jacob et al., 2017), including RPM1 (resistance to *Pseudomonas fluorescens*) (Bao et al., 2014), RPS2, and RPM (resistance to *Pseudomonas putida* in *Arabidopsis thaliana*) (Takahashi et al., 2003). In this omics analysis, although many HSPs were expressed, their corresponding protein expression levels were relatively low. A similar phenomenon was observed in *Arabidopsis thaliana* inoculated with *Pseudomonas syringae*, wherein the HSP90 gene was highly expressed, but the change in protein levels was not significant. According to a previous study, this may have been because plants synthesize SGT1 and HSP90 to cope with stress (Takahashi et al., 2003). In this study, the expression of SGT1 and RAR1 was detected simultaneously; however, the expression of RAR1 was not significantly different. Previous studies have shown that the expression of RAR1 is low under stress and does not respond strongly to pathogenic infection, whereas SGT1 is highly expressed upon infection or stress (Azevedo et al., 2006; Shirasu, 2009). This difference provides insights for a deeper understanding of plant disease resistance mechanisms mediated by HSPs.

CDPKs are serine/threonine protein kinases widely present in plants and protozoa, and they are important calcium (Ca²^+^) sensor proteins. In the plant immune defense system, Ca²^+^ signals are essential for both the PTI and ETI responses. CDPKs possess specific binding motifs, EF-hands, and an N-terminal catalytic kinase domain, which enables them to directly bind to Ca²^+^ or exert their functions by phosphorylating substrate proteins (Gao et al., 2014). Numerous studies have shown that the interactions between CDPKs and their target proteins or substrates can regulate various immune responses. These include the production of ROS during PTI and ETI, regulation of the expression of immune-related genes, and the hypersensitive response triggered by ETI (Freymark et al., 2007; Geng et al., 2013). For example, the wheat calcium-dependent protein kinase 2 (TaCDPK2) is a key protein involved in wheat resistance to powdery mildew. Moreover, the overexpression of TaCDPK2 in rice can significantly enhance the resistance to bacterial blight (Geng et al., 2013). Through transcriptomic and proteomic analyses, we observed the abundant transcripts and protein expression of CDPKs in alfalfa. Among them, three CDPK proteins (A0A072UWP5, A0A072V1D7, and G7J881) were significantly expressed after 13 days inoculation. These proteins were found to be involved in the plant-pathogen interaction (map04626) pathway. Based on the mechanisms of action of CDPKs, it is speculated that these proteins may be activated by Ca²^+^ signals when alfalfa is under pathogenic stress. By regulating downstream substrates, they participate in a complex defense response network and play a crucial role in the resistance of alfalfa to pathogens.

## 5 Conclusions

To elucidate the response mechanism of alfalfa to *F. acuminatum.* This study conducted a combined analysis of alfalfa inoculated with Ustilago maydis at 0, 3, and 13 days using transcriptomics and proteomics. After 3 days inoculation, DEGs and DEPs were mainly enriched in pathways related to the cell cortex, flavonoid biosynthesis, and amino acid metabolism. By day 13, the genes were primarily associated with the cell wall, defense response, and flavonoid biosynthesis.

The study also revealed that metabolic pathways, including secondary metabolites, flavonoids, glycolysis/gluconeogenesis, and plant-pathogen interactions, were significantly enriched at both the mRNA and protein levels, suggesting that these pathways play a key role in the alfalfa response to pathogen stress. During the defense process, the cell wall serves as the first barrier, and its key components, lignin and pectin, play critical roles. Genes involved in lignin biosynthesis and cell wall repair contribute to pathogen defense.

Additionally, antioxidant and defense-related enzymes (SOD, CAT, APX, and LOX) exhibited increased activity during the early stages of infection but decreased activity at later stages. These enzymes play essential roles in scavenging ROS and generating precursors for jasmonic acid synthesis, with LOX being particularly important in alfalfa resistance. HSPs and CDPKs were also significantly enriched. HSPs help protect plants from stress, whereas CDPKs regulate immune responses, with some potentially activated by Ca²^+^ signaling to modulate downstream defense mechanisms.

This study provides new insights into the molecular mechanisms underlying the response of alfalfa to *F. acuminatum* and offers a valuable basis for identifying candidate genes for breeding alfalfa that are resistant to root rot.

## Data Availability

The datasets presented in this study can be found in online repositories. The names of the repository/repositories and accession number(s) can be found below: https://www.ncbi.nlm.nih.gov/, PRJNA1256159 http://www.proteomexchange.org/, PXD053965.
