# Supplementary figures and images for: Transcriptomic–proteomic analysis reveals the regulatory mechanisms of Alfalfa (Medicago sativa) in response to Fusarium acuminatum
Source: Front Plant Sci. 2025 Jul 29;16:1620189. doi: 10.3389/fpls.2025.1620189 (PMC12351649; doi:10.3389/fpls.2025.1620189)

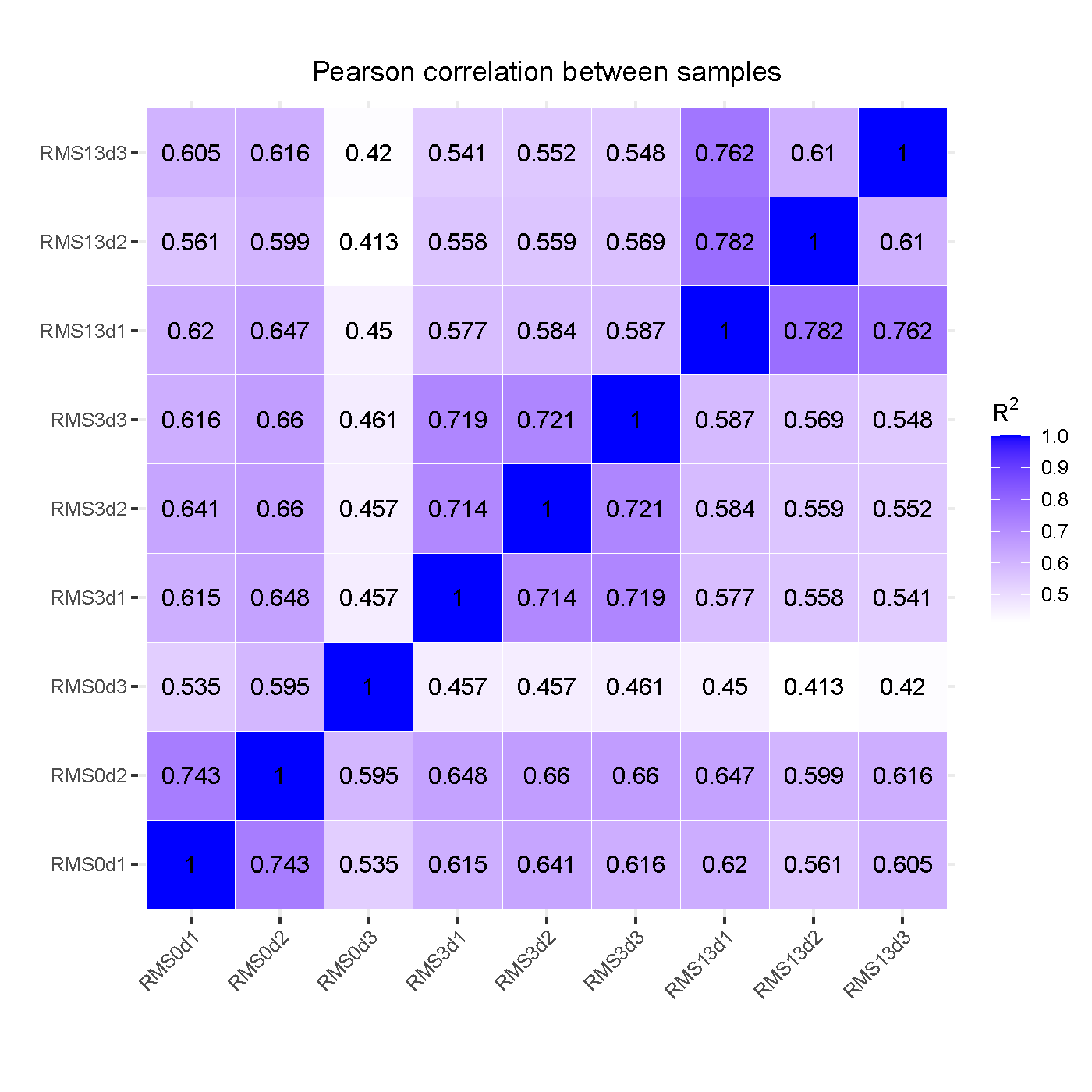

Supplement: Supplementary file 4 [file Image1.tiff]

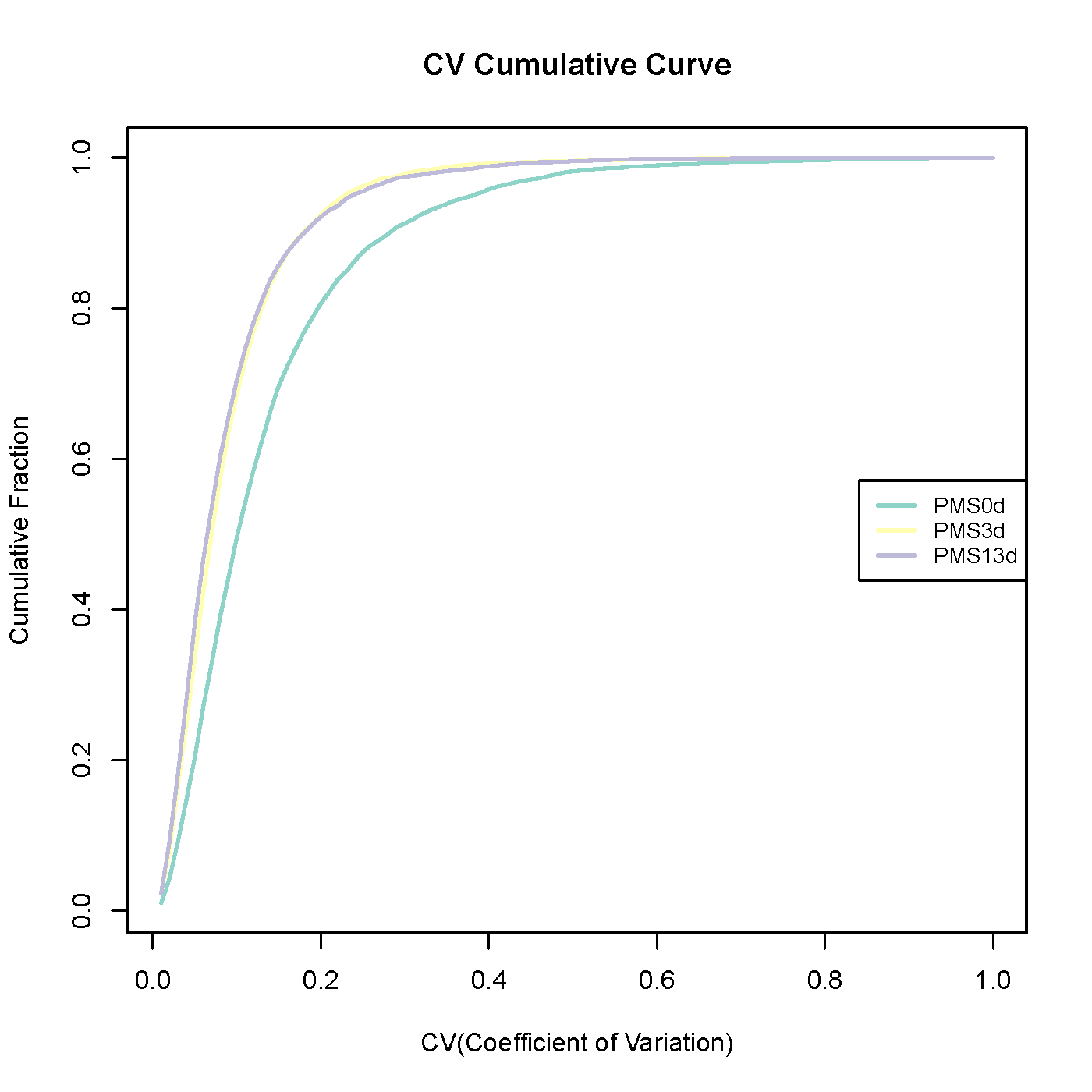

Supplement: Supplementary file 5 [file Image2.tiff]
